# VISUAL FUNCTIONS AND PERFORMANCE ON COGNITIVE CHALLENGE TEST IN OLDER ADULTS

**DOI:** 10.1093/geroni/igae098.3928

**Published:** 2024-12-31

**Authors:** Diane Zheng, Rosie Curiel, Alexandra Ortega, Kirsten Crenshaw, Denise Carballea, Brooke Bosworth, Emory Neer, David Loewenstein

**Affiliations:** University of Miami, Miami, Florida, United States; University of Miami, Miami, Florida, United States; University of Miami, Miami, Florida, United States; University of Miami, Miami, Florida, United States; University of Miami, Miami, Florida, United States; University of Miami, Miami, Florida, United States; University of Miami, Miami, Florida, United States; University of Miami, Miami, Florida, United States

## Abstract

The Loewenstein-Acevedo Scales for Semantic Interferences and Learning (LASSI-L) is a culturally sensitive Cognitive Challenge Test (CCT) that has been shown to be predictive of preclinical Alzheimer’s disease (AD). Visual impairment is a risk factor for AD and cognitive decline. Associations between LASSI-L and visual functions in older adults have never been evaluated. We assessed 133 adults aged 61 to 88 diagnosed with normal cognition, amnestic mild cognitive impairment (MCI), or pre-MCI using a comprehensive clinical evaluation and cognitive battery. The mean age was 73.5 years, 68% female, the average education was 15 years, and mean MMSE was 28.3. About 60% of the sample identified as Hispanic/Latino. Participants were administered objective vision tests measuring distance visual acuity and contrast sensitivity, and LASSI-L. Multiple linear regressions were performed to examine associations between visual function and LASSI-L measures. After adjusting for age, sex, and education, contrast sensitivity was statistically significantly associated with the LASSI-L maximum storage capacity (A2 cued recall, β=2.80, se=1.1, p=0.01), proactive semantic interference (PSI) (B1cued intrusions, β=-2.99, se=1.25, p=0.02), and failure to recover from proactive semantic interference (frPSI) (B2 cued recall, β=3.64, se=0.24, p=0.004). Distance visual acuity was not associated with LASSI-L measures. Contrast sensitivity, or the ability to discern characters on a similar color background or brightness, is an important visual function that facilitates daily activities. The association between contrast sensitivity and subtle cognitive decline such as PSI and frPSI on CCT suggests that visual changes co-occur during pre-dementia stages and may be considered alongside other diagnostic tools.

